# Association between Small Intestinal Bacterial Overgrowth and Subclinical Atheromatous Plaques

**DOI:** 10.3390/jcm12010314

**Published:** 2022-12-31

**Authors:** Changhao Dong, Guangxiang Wang, Rui Xian, Chao Li, Shaoxin Wang, Lihong Cui

**Affiliations:** 1 Department of Gastroenterology, School of Medicine, South China University of Technology, Guangzhou 510006, China; 2 Department of Gastroenterology, The Sixth Medical Center of PLA General Hospital of Beijing, Beijing 100048, China; 3 The Second School of Clinical Medicine, Southern Medical University, Guangzhou 510515, China

**Keywords:** gut flora, small intestine, dysbiosis, breath test, atherosclerosis

## Abstract

Background: Several recent studies have reported the relationship between atherosclerosis and gut microbial imbalance. Small intestinal bacterial overgrowth (SIBO) is one of the most common forms of gut microbiota imbalance, and studies have shown that SIBO plays an important role in human health. However, the relationship between SIBO and subclinical atheromatous plaques remains unclear. The aim of this study was to investigate the frequency of subclinical atheromatous plaques in patients with SIBO and to explore the association between these two conditions. Methods: A total of 411 eligible subjects were included in this study. The lactulose hydrogen-methane breath test was used to diagnose SIBO, and ultrasound examinations of the carotid, abdominal aorta and lower extremity arteries were performed in all subjects to assess the presence of plaques. Results: Plaques were more common in the SIBO-positive group than in the SIBO-negative group (abdominal aorta, 74.2% vs. 38.8%, *p* < 0.01; carotid arteries, 71.7% vs. 52.3, *p* < 0.01; lower extremity arteries, 73.4% vs. 57.6%, *p* < 0.01). After adjusting for traditional confounders, compared to the SIBO-negative population, the SIBO-positive population had, respectively, OR = 4.18 (95% CI = 2.56–6.80, *p* < 0.001), OR = 1.93 (95% CI = 1.23–3.02, *p* = 0.004), OR = 1.81 (95% CI = 1.14–2.88, *p* = 0.011) and OR = 5.42 (95% CI = 2.78–10.58, *p* < 0.001) for abdominal, carotid, lower extremity and any-territory plaque presence. Conclusion: SIBO was found to be associated with subclinical atheromatous plaques, and the mechanism of this association warrants further exploration.

## 1. Introduction

Cardiovascular disease, which includes hypertension, atherosclerosis, heart failure and a range of other diseases affecting the cardiovascular system, is one of the leading causes of the global disease burden [1]. Subclinical atherosclerosis is a state in which there is evidence of atherosclerotic plaque but no obvious clinical symptoms [2]. In-depth research on the nature of atherosclerosis, including its pathogenesis and the disease course evolution, has led to increased attention on the stage of subclinical atherosclerosis. The detection of subclinical atherosclerosis and appropriate early intervention is beneficial to reduce the risk of cardiovascular and cerebrovascular events.

The gut microbiota has become a hot research topic in the medical field in recent years, and it can independently influence or act as a biological barrier of the intestinal mucosa to participate in human metabolism and the development of various diseases. With the development of genome sequencing technologies and bioinformatics, a large body of literature supports the role of gut microbes in the occurrence and development of cardiovascular disease [3,4]. A recent study reported the results of shotgun sequencing of the gut microbiome in 218 patients with atherosclerotic cardiovascular disease (ACVD) and 187 healthy controls, and the investigators found that there was a significant imbalance in the composition and interspecies relationships of the gut microbiome in ACVD patients [5]. Small intestinal bacterial overgrowth (SIBO) is one of the most common forms of intestinal microbial dysbiosis, which refers to an alteration in the number or species of bacteria in the small intestine and causes a series of clinical symptoms. Studies have shown that SIBO is closely associated with several diseases, such as irritable bowel syndrome, nonalcoholic fatty liver disease, and heart failure [6,7,8,9]. According to the latest ACG clinical guidelines, a bacterial colony count of more than 10^3^ CFU/mL in small intestinal aspirates is considered the gold standard for the diagnosis of SIBO [10]. However, the time-consuming, expensive, and invasive characteristics of small bowel aspiration have limited its widespread application. The breath test is now a more common alternative method for the diagnosis of SIBO and is widely used in clinical practice [11]. To the best of our knowledge, to date, no study has attempted to assess the relationship between SIBO and subclinical atheromatous plaques. In this study, we used the hydrogen-methane breath test to diagnose SIBO and investigated the prevalence of carotid, abdominal aortic and lower extremity atheromatous plaques in SIBO-positive patients at the same time, aiming to explore the association between SIBO and subclinical atheromatous plaques.

## 2. Materials and Methods

This study was approved by the Ethics Committee of the Sixth Medical Center of PLA General Hospital and was conducted in accordance with the Declaration of Helsinki. Written informed consent was obtained from all subjects.

### 2.1. Study Population

This study was conducted at the Sixth Medical Center of PLA General Hospital from June 2021 to December 2021, and patients who visited the Gastroenterology Department for gastrointestinal discomfort were included in this study. The inclusion criteria were as follows: (1) age > 18 years and (2) no previous cardiovascular events (including stroke, myocardial infarction, angina pectoris, heart failure, coronary artery disease and atrial fibrillation). The exclusion criteria were as follows: (1) use of antibiotics or probiotics within the last four weeks; (2) colonoscopy within one week (3) postprandial hypoglycemia; (4) history of gastrointestinal surgery and (5) diagnosis of irritable bowel syndrome, inflammatory bowel disease, or chronic pancreatitis.

### 2.2. Lactulose Hydrogen-Methane Breath Test

A lactulose hydrogen methane breath test was performed on each patient included in the study after 8–12 h of fasting; the test was performed with reference to the North American Consensus [11]. To improve the accuracy of the test, patients were required to avoid fermentable foods such as complex carbohydrates the day before the test. Smoking and strenuous activity were prohibited on the day of the test.

The breath test instrument was purchased from QuinTron (Milwaukee, WI, USA). After measuring fasting H_2_ and CH_4_ levels, subjects were instructed to take 10 g of lactulose orally with one cup of water. Breath samples were collected every 30 min for a total time of 210 min. The diagnostic criteria for a positive SIBO were (1) H_2_ value rises ≥ 20 ppm from baseline by 90 min. (3) CH_4_ level ≥ 10 ppm during the test.

### 2.3. Assessment of Subclinical Atheromatous Plaques

Two-dimensional vascular ultrasound was performed on patients to assess the presence of subclinical atherosclerotic plaques in the abdominal aorta, carotid arteries, and lower extremity arteries (including common femoral, deep femoral, superficial femoral, and popliteal arteries). Plaques were defined according to the Mannheim consensus as focal structures protruding into the artery lumen of at least 0.5 mm, or 50% of the surrounding IMT value, or a thickness more than 1.5 mm measured from the media-adventitia interface to the intima-lumen interface [12]. Patients were classified as having a plaque or not, regardless of severity.

### 2.4. Clinical Measurements

Anthropometric and biochemical evaluations were performed for all patients in the fasting state. Clinical data were recorded including age, sex, medical history, medication, smoking, alcohol consumption, weight, height, body mass index (BMI), and blood pressure. Each patient’s gastrointestinal symptoms were also questioned and recorded in detail (including food allergy or intolerance, belching, dyspepsia, abdominal pain, constipation, diarrhea, alternating constipation and diarrhea, bloating, intestinal exhaust bowel, loss of appetite, tenesmus, and nausea). Serum biochemical examinations including fasting glucose (FG), total cholesterol (TC), triglyceride (TG), high-density lipoprotein cholesterol (HDL-C) and low-density lipoprotein cholesterol (LDL-C) were evaluated by an automatic biochemical analyzer.

### 2.5. Statistical Analysis

Continuous variables are presented as the means ± standard deviations, and categorical variables are expressed as frequencies and percentages. Student’s t-test, Mann–Whitney U test and chi-square test were used for comparisons between groups. Multiple logistic regression models were constructed to identify risk factors associated with atherosclerotic plaques in the carotid arteries, abdominal aorta, lower extremity arteries, or any of the three territories. The results of the logistic analysis are expressed as odds ratios (ORs) and 95% confidence intervals (95% CIs). *p* < 0.05 was considered indicative of statistical significance. All statistical analyses were performed using SPSS version 26.0 (IBM, Armonk, NY, USA). 

## 3. Results

A total of 411 patients were included in this study (mean age, 59.2 years; range, 28–85 years), of whom 241 (58.6%) were diagnosed as SIBO positive and 170 (41.4%) as SIBO negative. The comparative characteristics of SIBO-positive and SIBO-negative patients are shown in Table 1. The mean age of the SIBO-positive group was greater than that of the SIBO-negative group (60.9 vs. 56.6, *p* < 0.01). The frequencies of patients in the SIBO-positive group with a plaque in the abdominal aorta, carotid artery, and lower extremity arteries were 179, 173, and 177, respectively. For each territory, the prevalence of plaqueswas significantly higher in the SIBO-positive group than in the SIBO-negative group, and the differences were statistically significant. There were no significant differences between the two groups in terms of sex, BMI, smoking history, alcohol consumption, laboratory data, or prevalence of hypertension and diabetes.

**Figure 1:**
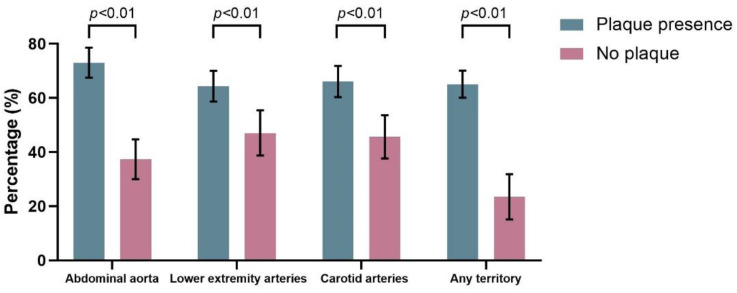
The prevalence of SIBO.

**Figure 2:**
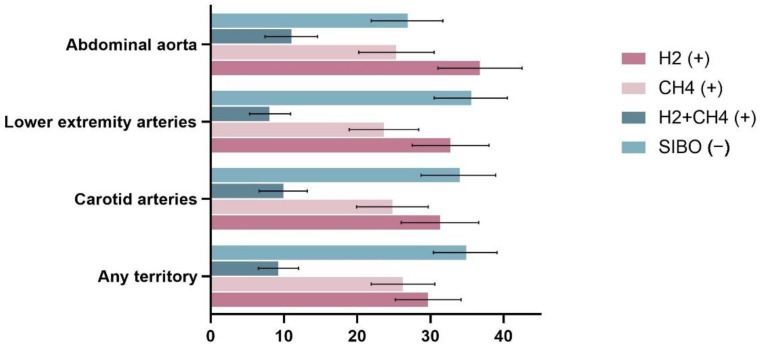
Proportion of SIBO subtypes among the populations with plaques.

The prevalence of SIBO is shown in Figure 1. SIBO was found in 179/245 patients with abdominal aortic plaque compared to 62/166 who did not have abdominal aortic plaque (73.1% vs. 37.3%, *p* < 0.01). Among 411 patients, 275 had at least one arterial plaque in the lower extremities, and SIBO was substantially more common in those with lower extremity plaques (64.3% vs. 47.0%, *p* < 0.01). The prevalence of SIBO was significantly higher in those with a carotid plaque than in those without carotid plaque (66.0% vs. 45.6%, *p* < 0.01). Overall, the prevalence of SIBO was significantly higher in those with a plaque in any of the above three territories than in those without any plaque (65.0% vs. 23.4%, *p* < 0.01). Moreover, we also analyzed the subtypes of SIBO in different groups (Figure 2). The results showed that in all four groups above, the prevalence of H2-positive was significantly higher in patients with plaques than in those without plaque (*p* < 0.05). However, only in the abdominal aorta and the any-territory group did we observe a higher prevalence of CH4-positive in patients with plaques than in those without (*p* < 0.05).

As shown in Table 2, bloating was the most common of all investigated symptoms (248/411, 60.3%). Among the symptoms investigated, bloating (*p* < 0.01), loss of appetite (*p* < 0.01), and food allergy or intolerance (*p* < 0.05) were significantly more prevalent in the SIBO-positive group than in the SIBO-negative group. There was no significant difference in the prevalence of other symptoms between the two groups.

Logistic regression analysis (Table 3) showed that SIBO was significantly associated with the presence of subclinical atheromatous plaques in all three territories, and this association remained significant after adjusting for potential confounders (abdominal aorta, OR = 4.18, 95% CI = 2.56–6.80, *p* < 0.001; carotid arteries, OR = 1.93, 95% CI = 1.23–3.02, *p* = 0.004; lower extremity arteries, OR = 1.81, 95% CI = 1.14–2.88, *p* = 0.011). For any of the three territories, the odds of having a subclinical atheromatous plaque were 5.42 times higher in the SIBO-positive population than in the SIBO-negative population (95% CI = 2.78–10.58, *p* < 0.001).

## 4. Discussion

In the present study, we found a higher prevalence of subclinical atheromatous plaques in the SIBO-positive population than in the SIBO-negative population. Multiple logistic regression analysis showed that the difference was still significant after adjusting for traditional risk factors, which indicated an association between SIBO and subclinical atheromatous plaques.

Atherosclerosis is a complex disease involving both inflammation and metabolism and has traditionally been considered a lipid-driven disease. However, recently, with the emergence of substantial new evidence, the gut microbiota has gained widespread attention as one of the non-traditional drivers of cardiovascular disease [13]. Fecal microbiota transplantation in animal research provides evidence of a causal relationship between intestinal microbiota and atherosclerosis [14]. For example, Brandsma and his colleagues transplanted pro-inflammatory microbiota from Caspase1^−/−^ mice into Ldlr^−/−^ (low-density lipoprotein receptor knockout) mice and the results showed that the transplanted mice had 29% larger atherosclerotic areas and significantly increased levels of inflammation than control mice [15]. Alternatively, in addition to inflammation, some intestinal metabolites are recognized to contribute to the formation of atherosclerosis [16]. Both human and animal studies have shown that gut microbes can metabolize some nutrients in food (e.g., choline, carnitine, phosphatidylcholine) to form trimethylamine-N-oxide (TMAO), a substance involved in the development of atherosclerosis [17]. Several recent studies have indicated that TMAO not only influences the traditional risk factors for atherosclerosis but also plays an important role in the pathophysiology of atherosclerosis as an independent factor [18]. SIBO is a disease characterized by dysbiosis of the gut microbiota, shown as an increased bacterial load in the small intestine. The breath test is based on the measurement of hydrogen and methane levels produced by intestinal bacteria fermenting substrates such as lactulose and is currently the most common method used to diagnose SIBO. Recently, Mollar et al. [19] reported for the first time the association between SIBO and TMAO, and they found a significant positive correlation between TMAO levels and exhaled hydrogen concentration in patients with heart failure.

Only one study to date has reported an association between SIBO and subclinical atherosclerosis. Ponziani et al. [20] found a reduced activation of matrix GLA-protein (MGP) in the plasma of patients with SIBO, which provides an idea for the possible mechanism of association between SIBO and subclinical atherosclerosis. MGP is a vitamin K-dependent protein that helps prevent calcium from accumulating in the arterial walls and is associated with the signs of early vascular disease and the development of cardiovascular disease. The study also compared carotid intima-media thickness (cIMT) and local pulse-wave velocity (PWV) in patients with and without SIBO and found that the PWV values were significantly higher in SIBO-positive patients than in SIBO-negative patients. However, the study has certain points of limitations, first is that the total number of patients in the study was only 39, and the small sample size might limit the power of it. Secondly, the study did not include traditional cardiovascular risk factors for multivariate analysis. In addition, although PWV and cIMT are known to be markers for the early detection and risk prediction of subclinical atherosclerosis [21,22], some studies have shown that the assessment of carotid plaques seems to be more meaningful than cIMT in detecting coronary artery disease and predicting cardiovascular events [23,24]. Moreover, PWV does have limitations in the assessment of subclinical atherosclerosis. There are many clinical factors that can influence PWV values, such as age, blood pressure, and other cardiovascular risk factors, with blood pressure in particular being the largest confounder in PWV interpretation [21]. To the best of our knowledge, the present study is the first to assess the association between SIBO and subclinical atheromatous plaques. Our study has investigated plaques in three territories including carotid, abdominal aorta and lower extremity arteries, and incorporated traditional cardiovascular risk factors for multivariate analysis.

According to the comparison of baseline data, the SIBO-positive group was older than the SIBO-negative group (*p* < 0.01), which is consistent with previous studies. Older age is closely associated with SIBO [25], which may be related to the fact that older adults are more likely to have a combination of risk factors for SIBO, such as decreased intestinal motility, small bowel diverticulosis, intestinal surgery and achlorhydria, as well as higher medication use. Moreover, patients with SIBO often have some non-specific symptoms, but the exact mechanism by which these symptoms occur is not well understood [26]. In our study, we found that symptoms including bloating, loss of appetite and food intolerance were more common in patients with SIBO, which we speculate may be related to excessive gas production as a result of the abnormal luminal fermentation of intestinal bacteria. In addition, it is known that the bacteria in SIBO may significantly interfere with the enzymatic, absorptive and metabolic actions of the human body, and SIBO is thought to be associated with maldigestion and malabsorption [27], which may also contribute to the development of symptoms.

Although the mechanism of the association between SIBO and atherosclerosis is unknown, chronic inflammation may be one of the reasons. This chronic inflammation in SIBO patients may be associated with an impaired intestinal barrier and increased permeability caused by bacterial overload [28,29]. The physical barrier of the small intestine is mainly composed of epithelial cells and tight junctions, and this structural feature makes it easier for exogenous peptides as well as bacterial components to penetrate into the internal environment. Under the condition of SIBO, increased bacterial adhesion and luminal endotoxin can lead to an alteration in gut permeability, thus facilitating the entry of endotoxin produced by the already enlarged bacterial pool into the bloodstream and activating systemic inflammatory and immune responses. In addition, because of the presence of colon-specific bacteria in the small intestine, the deconjugation of bile salts occurs earlier in the small intestine, which not only disrupts the digestion of fats, but the increased free bile acids also have a direct toxic effect on the intestinal mucosa, leading to aggravated damage of the intestinal mucosal barrier [30].

Malabsorption of vitamins in SIBO patients is also a topic of interest. Cobalamin (vitamin B12) deficiency appears to occur commonly in SIBO patients as a result of increased vitamin B12 use by excess anaerobic bacteria, and the absorption of vitamin B12 is also significantly reduced in SIBO patients due to the competitive uptake by luminal bacteria [27]. Vitamin B12 deficiency is known to potentially lead to an increase in homocysteine levels, and elevated plasma homocysteine is an important risk factor for cardiovascular disease [31,32]. A randomized controlled trial from Hodis et al. [32] showed that high-dose supplementation with B vitamins can reduce the progression of early subclinical atherosclerosis. Furthermore, our study showed that plaques seem to be more closely associated with the overgrowth of hydrogen-producing bacteria (Figure 2). A recent study by Madigan et al. [33] found that the overgrowth of hydrogen-producing bacteria was more likely to lead to a reduction in vitamin B12 than the overgrowth of methanogenic Archaea, which may be related to the fact that methanogenic bacteria in the human gut possess the metabolic machinery for synthesizing vitamin B12. Therefore, we speculate that vitamin B12 deficiency in SIBO patients, especially those with hydrogen-producing bacterial overgrowth, may be one of the possible mechanisms for its association with atheromatous plaques, but further studies are needed to confirm this.

The present study has several limitations. First, the study population was limited to patients with gastrointestinal discomfort, and the conclusions may not be extrapolated to all populations. We suggest further multicenter studies that include healthy populations, which would be beneficial for the generalization of the results. Second, the cross-sectional analysis did not allow us to establish a causal relationship between SIBO and subclinical atheromatous plaque, and we were unable to determine the timing and sequence of the occurrence of SIBO and atheromatous plaques. Last, breath testing is not the gold standard for diagnosing SIBO, but it has an acceptable level of accuracy and is widely used in clinical practice and scientific research.

## 5. Conclusions

In summary, in the present study, we found that SIBO was associated with a higher prevalence of subclinical atheromatous plaques in the three territories we investigated (including carotid, abdominal aorta, and lower extremity arteries) and was independent of other traditional cardiovascular risk factors, but it should be noted that the conclusion is limited to a population of patients with gastrointestinal symptoms. Further prospective and multicenter studies are needed to confirm this association as well as to determine whether the treatment of SIBO has a positive effect on slowing the progression of atherosclerosis.

## Figures and Tables

**Table 1:** Characteristics of demographic, biochemical, clinical and plaque presence in SIBO-positive and SIBO-negative groups.

	Overall (*n* = 411)	SIBO-Negative (*n* = 170)	SIBO-Positive (*n* = 241)	*p*-Value
Age	59.2 ± 9.9	56.6 ± 10.5	60.9 ± 9.1	<0.01
Gender				0.20
male	246 (59.8)	108 (63.5)	138 (57.3)	
female	165 (40.2)	62 (36.5)	103 (42.7)	
BMI (kg/m^2^)	24.7 ± 3.2	25.0 ± 3.6	24.5 ± 2.9	0.10
Smoking	97 (22.8)	41 (24.1)	53 (21.9)	0.61
Alcohol	164 (39.9)	70 (41.1)	94 (39.0)	0.66
Hypertension	135 (32.8)	62 (36.4)	73 (30.2)	0.19
Diabetes	71 (17.2)	34 (20.0)	37 (15.3)	0.23
FG (mmol/L)	5.7 ± 1.2	5.8 ± 1.3	5.7 ± 1.2	0.36
HDL-C (mmol/L)	1.3 ± 0.4	1.3 ± 0.3	1.3 ± 0.4	0.62
LDL-C (mmol/L)	2.9 ± 0.8	2.9 ± 0.8	2.9 ± 0.8	0.40
Triglycerides (mmol/L)	1.8 ± 1.8	2.0 ± 2.2	1.7 ± 1.4	0.11
Total cholesterol (mmol/L)	4.9 ± 1.1	5.0 ± 1.2	4.8 ± 1.1	0.34
Atheromatous plaque				
Abdominal aorta	245 (59.6)	66 (38.8)	179 (74.2)	<0.01
Carotid arteries	262 (63.7)	89 (52.3)	173 (71.7)	<0.01
Lower extremity arteries	275 (66.9)	98 (57.6)	177 (73.4)	<0.01

BMI, body mass index; SIBO, small intestinal bacterial overgrowth; FG, fasting glucose; HDL-C, high-density lipoprotein cholesterol; LDL-C, low-density lipoprotein cholesterol.

**Table 2:** Comparison of gastrointestinal symptoms between the SIBO-positive and SIBO-negative groups.

	Overall (*n* = 411)	SIBO-Negative (*n* = 170)	SIBO-Positive (*n* = 241)	*p*-Value
Food allergy or intolerance	79 (19.2)	24 (14.1)	55 (22.8)	0.03
Belching	196 (47.7)	79 (46.5)	117 (48.5)	0.68
Dyspepsia	176 (42.8)	74 (43.5)	102 (42.3)	0.81
Abdominal pain	150 (36.5)	57 (33.5)	93 (38.6)	0.29
Constipation	148 (36.0)	61 (35.9)	87 (36.1)	0.96
Diarrhea	93 (22.6)	43 (25.3)	50 (20.7)	0.28
Alternating constipation and diarrhea	79 (19.2)	30 (17.6)	49 (20.3)	0.50
Bloating	248 (60.3)	87 (51.2)	161 (66.8)	<0.01
Intestinal exhaust	214 (52.1)	81 (47.6)	133 (55.2)	0.13
Loss of appetite	102 (24.8)	27 (15.9)	75 (31.1)	<0.01
Tenesmus	106 (25.8)	37 (21.8)	69 (28.6)	0.12
Nausea	62 (15.1)	30 (17.6)	32 (13.3)	0.22

**Table 3:** Odds ratios (95% CIs) for plaque presence in different territories of the SIBO-positive and SIBO-negative groups.

	SIBO-Negative	SIBO-Positive	*p*-Value
Abdominal aorta			
model 1	1.00 (ref)	4.55 (2.98–6.94)	<0.001
model 2	1.00 (ref)	4.21 (2.59–6.82)	<0.001
model 3	1.00 (ref)	4.18 (2.56–6.80)	<0.001
Carotid arteries			
model 1	1.00 (ref)	2.32 (1.53–3.49)	0.003
model 2	1.00 (ref)	1.80 (1.16–2.80)	0.009
model 3	1.00 (ref)	1.93 (1.23–3.02)	0.004
Lower extremity arteries			
model 1	1.00 (ref)	2.03 (1.34–3.09)	0.001
model 2	1.00 (ref)	1.64 (1.05–2.57)	0.031
model 3	1.00 (ref)	1.81 (1.14–2.88)	0.011
Any territory			
model 1	1.00 (ref)	6.10 (3.29–11.33)	<0.001
model 2	1.00 (ref)	4.76 (2.48–9.14)	<0.001
model 3	1.00 (ref)	5.42 (2.78–10.58)	<0.001

Model 1 is unadjusted; Model 2 is adjusted for age and sex; Model 3 is adjusted for age, sex, BMI, smoking, alcohol consumption, hypertension, diabetes, fasting glucose, high density lipoprotein cholesterol, low density lipoprotein cholesterol, triglycerides and total cholesterol.

## Data Availability

The data analyzed in this study can be obtained on reasonable request from the corresponding author.
